# CSID: A Novel Multimodal Image Fusion Algorithm for Enhanced Clinical Diagnosis

**DOI:** 10.3390/diagnostics10110904

**Published:** 2020-11-05

**Authors:** Shah Rukh Muzammil, Sarmad Maqsood, Shahab Haider, Robertas Damaševičius

**Affiliations:** 1 Department of Computer Science, City University of Science and Information Technology, Peshawar 25000, Pakistan; kashafshah904@gmail.com (S.R.M.); dr.shahab39@gmail.com (S.H.); 2 Department of Software Engineering, Kaunas University of Technology, Kaunas 51368, Lithuania; sarmad.maqsood@ktu.edu

**Keywords:** medical image processing, image fusion, multimodal medical image, image decomposition, sparse representation

## Abstract

Technology-assisted clinical diagnosis has gained tremendous importance in modern day healthcare systems. To this end, multimodal medical image fusion has gained great attention from the research community. There are several fusion algorithms that merge Computed Tomography (CT) and Magnetic Resonance Images (MRI) to extract detailed information, which is used to enhance clinical diagnosis. However, these algorithms exhibit several limitations, such as blurred edges during decomposition, excessive information loss that gives rise to false structural artifacts, and high spatial distortion due to inadequate contrast. To resolve these issues, this paper proposes a novel algorithm, namely Convolutional Sparse Image Decomposition (CSID), that fuses CT and MR images. CSID uses contrast stretching and the spatial gradient method to identify edges in source images and employs cartoon-texture decomposition, which creates an overcomplete dictionary. Moreover, this work proposes a modified convolutional sparse coding method and employs improved decision maps and the fusion rule to obtain the final fused image. Simulation results using six datasets of multimodal images demonstrate that CSID achieves superior performance, in terms of visual quality and enriched information extraction, in comparison with eminent image fusion algorithms.

## 1. Introduction

Image processing manipulates input source images to extract the maximum possible information. The information obtained is exploited for several applications, including remote sensing, malware analysis, clinical diagnosis, etc. [1,2,3,4,5]. However, the latter requires greater attention as enhanced clinical diagnosis remains the top priority around the world [6]. In this regard, clinical imaging plays a vital role in modern day health care systems, where Computed Tomography (CT) and Magnetic Resonance Imaging (MRI) are among the most extensively used imaging modalities [7,8,9]. This allows radiologists to analyze the human body and generate different patterns, which are used in clinical analysis [10]. These images provide anatomical statistics [7]; however, the extraction of purposeful functional details from an individual image remains a critical issue. This demands multimodal image fusion, which integrates the complementary information of images from different modalities to produce an enhanced fused image through simulation, thereby providing enriched anatomical and functional information [6,7,11,12,13].

There are several multimodal image fusion algorithms [7,14,15], which are divided into two major classes, namely the spatial and transform domains [2]. The spatial domain produces fused images based on the coefficient deduced from pixels, sections, or blocks without transformation [14,16], which produces false structural artifacts in the resulting fused images. Conversely, the transform domain merges the respective transform coefficients and employs the inverse transformation to yield better fused images. To this end, MultiScale Transformation (MST) includes contourlet transform [17], discrete wavelet transform [18], non-subsampled contourlet transform [19], and curvelet transform [20], which are used for multimodal image fusion.

There are a number of the MST-based algorithms. For example, the authors in [18] proposed a novel Discrete Wavelet Transform-based (DWT) algorithm. The proposed algorithm selects the low frequency domain coefficients by employing the maximum sharpness focus measure method, whereas the high frequency sub-band coefficients are chosen on the basis of maximum neighboring energy-based fusion. Similarly, Non-Subsampled Contourlet Transform (NSCT) [19] decomposes the input source images into a series of high frequency sub-bands and one low frequency sub-band. The work in [19] also proposed activity measures for low-pass and high-pass sub-bands to enhance fusion. The sub-bands obtained from the aforementioned process are merged based on the corresponding activity measure. Finally, the inverse NSCT is applied on the merged sub-bands to build a fused image. Furthermore, the Laplacian Pyramid (LP) [21] decomposes the source images into different low-pass filtered images and produces a pyramid structure, where the visual quality of the resulting image remains proportional to the pyramid levels. Moreover, Guided Filtering-based Fusion (GFF) [22] and Convolutional Sparse Representation (CSR) [23] decompose the images into base and detail layers. GFF aims to enhance the spatial consistency of fused images, whereas CSR preserves the detailed information from the input source images. Additionally, Convolutional Sparsity-based Morphological Component Analysis (CSMCA) [24] integrates MCA and CSR into a a novel optimization framework to enhance the fusion process. Another algorithm, namely Convolutional Neural Network (CNN) [25] adopts the Siamese convolutional network to create weight maps. These weight maps integrate pixel activity information from the input source images and employ the local similarity-based strategy to adapt fusion modes for the decomposed coefficients. However, the aforementioned algorithms exhibit several limitations, such as the blurring effect near strong edges during image decomposition and increased information loss, which impact the originality of the fused images, thereby producing high spatial distortion.

To resolve the aforementioned issues, we propose a novel algorithm for multimodal image fusion, namely Convolutional Sparse Image Decomposition (CSID), having the following contributions.

1. We employ contrast stretching and the spatial gradient method to extract edges from the input source images.
2. We propose the use of the cartoon-texture decomposition that creates an over-complete dictionary.
3. We propose a modified Convolutional Sparse Coding (CSC) method.
4. Finally, our proposed algorithm uses enhanced decision maps and a fusion rule to obtain the fused image.
5. Additionally, this simulation study reveals that the CSID algorithm achieves superior performance, in terms of visual quality and enriched information extraction, in comparison with other image fusion algorithms, as it will be discussed in Section 5.

The rest of the paper is organized as follows. Section 2 critically reviews the eminent related work on multimodal image fusion. Section 3 details the proposed algorithm. Section 4 presents the objective evaluation metrics. Section 5 evaluates the performance of the suggested algorithm in comparison with the state-of-the-art eminent algorithms. Finally, Section 6 concludes the paper with discussion on future research aims.

## 2. Related Work

Modern healthcare systems actively use multimodal image fusion for diagnosis [10]. This section critically reviews the eminent work on multimodal clinical image fusion.

Recently, the MST and Sparse Representation (SR) techniques have gained significant popularity in the transform domain and have produced positive results in medical image analysis [26]. However, these methods have shortcomings, such as (i) the “max-*l*1” rule induces spatial inconsistency in a fused image when different modalities are captured from the source images [27], (ii) the MST-based filters used for the SR-based image fusion [28] are time-dependent due to the training of dictionary and its optimization, and (iii) these algorithms are also unable to decompose several types of images [12]. Another challenge is the complicated oriented shape of source images that cannot be precisely categorized through an already trained dictionary [28]. To address these issues, the authors in [29] propose a training model that employs the well-known K-means algorithm [30]. Research in the domain of multimodal image fusion have produced promising outcomes; however, there are several drawbacks. The authors in [15,25] propose neural network-based fusion algorithms that efficiently adjust and fit the training parameters, but these algorithms are not capable of representing information from multiple sources [31].

Moreover, learning-based algorithms are also found to be useful in multimodal image fusion [32]. To this end, SR in combination with learning-based multimodal medical image fusion strategies are gaining interest of the research community [32]. The works in [23,27,33] employ the SR-based algorithms for image fusion. Similarly, the authors in [34] propose enhanced sparse representation orthogonal matching pursuit (SR-OMP) algorithms. Furthermore, the work in [24] presents another SR-based morphological component analysis model for pixel level image fusion. However, the blurring effect during decomposition restricts the performance of the proposed model. The authors in [35] present a multimodal image fusion algorithm that employs the SR-based cartoon-texture decomposition. However, it also faces the blurring issue during decomposition that results in considerable information loss. Additionally, the pyramid transformation [36] algorithm exhibits limited performance due to inaccuracy in information capturing and path details. Arif et al. [37] present a multimodal image fusion algorithm based on the curvelet transform and genetic algorithm (GA). Here, GA solves the suspicions and evaluates an optimized fused image. Kaur et al. [38] propose another image fusion algorithm that employs deep belief networks. Maqsood et al. [2] present a two scale image decomposition technique, where the spatial gradient-based edge detection method is used to acquire the detail layer and the SR rule is used to construct a fused image. This method produces improved image fusion results, however, it still experiences false structured artifacts in the fused image. Shahdoosti et al. [39] propose a sparse representation in the tetrolet domain for medical image fusion; however, this approach falls a victim of overlapping artifacts in the fused images.

From the literature survey, it is found that the SR-based image fusion algorithms have the advantage of better information extraction in comparison with other fusion algorithms. However, there are several issues that require urgent attention, such as (i) blurring effect near strong edges during image decomposition, (ii) appearance of false structured artifacts in fused image, and (iii) reduced contrast that results in high spatial distortion. To resolve these issues, we propose a novel image fusion algorithm that is detailed in the following section.

## 3. The Proposed Convolutional Sparse Image Decomposition (CSID) Algorithm

**Figure 1:**
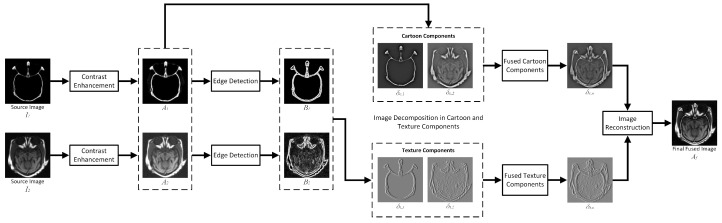
Procedural flowchart of the proposed CSID algorithm.

This section presents our proposed novel algorithm for multimodal image fusion, namely, Convolutional Sparse Image Decomposition (CSID). CSID comprises six phases that include contrast enhancement, edge detection, cartoon and texture decomposition, enhanced CSC-based sparse coding, sparse coefficient maps fusion, and fused image reconstruction, as depicted in Figure 1. These phases are detailed in the following subsections.

We take a source image ($I_{i}$) having *P* × *Q* dimensions, where P=1,2,3,…,p, Q=1,2,3,…,q, and i∈[1,2] that represents the CT and MRI images, respectively. CSID starts with contrast enhancement, which is detailed in the following subsection.

### 3.1. Contrast Enhancement

Contrast enhancement is a major preprocessing step in image fusion for diagnostics processes [40]. No-Reference Image Quality Assessment (NR-IQA) and Non-Parametric Histogram Equalization (NPHE) are commonly used for contrast enhancement [41,42]. NR-IQA employs histogram equalization and uses structural-similarity index metric to generate images with enhanced contrast, whereas NPHE employs modified spatial transformation-based adaptive contrast enhancement. However, these techniques require manual parameter tuning that limits their performance in accurately reflecting the image contrast with respect to an input image. To resolve the aforementioned issues, CSID employs the Bio Inspired Multi Exposure Fusion (BIMEF) framework [40] that improves contrast and preserves mean brightness of the source images. BIMEF uses illumination estimation for the construction of a weighted matrix. Thus, we start with the detection of optimal exposures using a camera response model, thereby, producing synthetic images that are better exposed in the regions in comparison with source images. Furthermore, we apply the weight matrix, with an appropriate exposure, upon the synthetic image, which is then merged with the source image for contrast enhancement. Here, to conserve the contrast of an image, a weighted matrix is associated with the scene brightness that is computed as follows [40],

(1) $$\overset{´}{I}=A^{\phi },$$

where *A* denotes the scene brightness map and ϕ is a parameter managing the degree of enhancement. Moreover, since the highest regional maxima is better identified using a max function in comparison with a min function [43,44], CSID computes the dark regions (*R*), based upon initial estimation of brightness for each pixel (*x*), as [40],

(2) $$R\left(p,q\right)=\max\overset{´}{I}.$$

Since absolute brightness has local consistency for the boundaries with same structures, *A* eliminates the textural edges and builds a significant structure of an image. CSID optimizes *A* as [40],

(3) $$X\left(p,q\right)=\min_{A}||A-{R||}_{2}^{2}+\varphi ||W\circ ▽{A||}_{1},$$

where $\left|\right|★{\left|\right|}_{1}$ and $\left|\right|★{\left|\right|}_{2}$ represent the $l_{1}$ and $l_{2}$ norms, respectively, ▽ is the first order derivative filter consisting ${▽}_{h}$*A* and ${▽}_{v}$*A* as horizontal and vertical components, respectively. φ denotes the coefficient and *W* refers to the weighted matrix. The weighted matrix is further refined to obtain significant edges in an image as [40],

(4) $$Z_{d}\left(p,q\right)=\frac{1}{|\sum_{y\epsilon d(p,q)}{▽}_{f}X\left(p,q\right)|+\kappa },j\in \left(h,v\right),$$

where |★| denotes the positive value operator, d(p,q) is the neighborhood window pointed at pixels *p* and *q*, and κ represents the constant to evade zero denominator. Moreover, we use (5) to evaluate $A_{i}$ that minimizes complexity. $A_{i}$ is then applied upon the source image to generate the final outcome of this phase, i.e., an image with enhanced contrast.

(5) $$A_{i}=\min_{A}\sum_{p,q}\left({\left(A\left(p,q\right)-d\left(p,q\right)\right)}^{2}+\varphi \sum_{j\epsilon (h,v)}\frac{Z_{d}\left(p,q\right)({▽}_{f}{\left(A\left(p,q\right)\right)}^{2}}{|{▽}_{f}X\left(p,q\right)|+\epsilon }\right).$$

**Figure 2:**
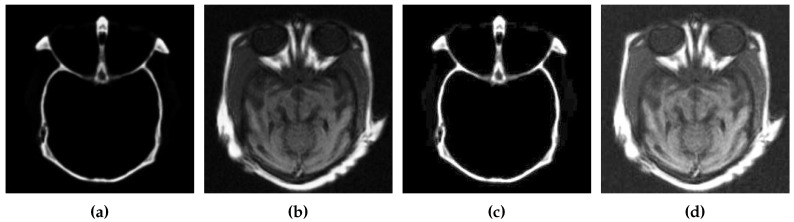
Contrast enhancement results, where (**a**,**b**) represent the source images and (**c**,**d**) show images with enhanced contrast using BIMEF.

The results of contract enhancement are illustrated in Figure 2. After contrast enhancement, CSID enters the second phase, which is detailed in the following subsection.

### 3.2. Edge Detection

**Figure 3:**
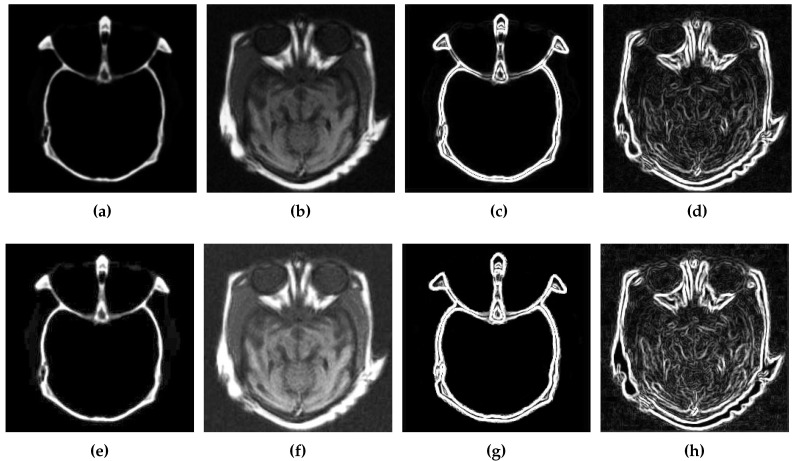
Edge detection results, where (**a**,**b**) are the original source images, (**c**,**d**) represent the gradients of (**a**,**b**) using the Sobel method, (**e**,**f**) shows the images with enhanced contrast using BIMEF, and (**g**,**h**) represent the gradients of (**e**,**f**) using the Sobel method.

Edge detection finds the boundaries of objects in an image through identification of brightness discontinuities [45]. To this end, CSID performs edge detection in the image, obtained after contrast enhancement, by employing the Sobel operator [45]. Edge detection yields better performance when applied upon the images with enhanced contrast in comparison with original source images, as it is demonstrated in Figure 3.

For edge detection, CSID includes the image gradient approximation, where each location is either the corresponding gradient vector or the norm of this vector. The image is convoluted with the first kernel from left to right and the gradient for the *X* coordinate is obtained as,

(6) $$B_{p}=\left[\begin{array}{ccc} 1 & 0 & -1\\ 2 & 0 & -2\\ 1 & 0 & -1 \end{array}\right].$$

Similarly, the gradient for the *Y* coordinate is obtained by convoluting the first kernel from top to bottom as,

(7) $$B_{q}=\left[\begin{array}{ccc} 1 & 2 & 1\\ 0 & 0 & 0\\ -1 & -2 & -1 \end{array}\right].$$

Furthermore, the image gradient vectors, obtained using (6) and (7), are used to find edges as,

(8) $$B_{i}=\sqrt{{\left(B_{p}\right)}^{2}+{\left(B_{q}\right)}^{2}}.$$

Figure 3 depicts a comparison of edge information in the source and enhanced images obtained after completion of the first two phases of our proposed CSID algorithm. Figure 3a,b present the source CT and MRI images, respectively, while their edge maps are demonstrated in Figure 3c,d, respectively. Furthermore, Figure 3e,f include the improved CT and MRI images obtained after contrast enhancement (as detailed in Section 3.1) and their respective gradient maps are shown in Figure 3g,h. Here, improved edge detection is observed in the image with an enhanced contrast in comparison with edge detection in the original source image. On completion of this phase, CSID proceeds to the third phase, which is discussed in the following subsection.

### 3.3. Cartoon and Texture Decomposition

Cartoon-texture decomposition divides an image into the geometric cartoon and texture components, which removes the background interference. To this end, we propose a modification to the legacy Convolutional Sparse Coding (CSC) [46]. In our proposed modified CSC model, a similarity threshold is maintained to compute residual correlation similarity between the selected sparsest coefficients and the other coefficients in the sparse coding phase, which is discussed in Section 3.4. This expands the coefficients set, thereby, obtaining more suitable coefficients for SR. Furthermore, it also accelerates the process of sparse coding, and the residual correlation similarity easily minimizes the target error for each image patch signal. Moreover, multiple similar patches are used to represent a single image patch that further enhances the fused image by avoiding the blurring effect. An optimized solution of the CSC problem using (9) is generated as,

(9) $$\begin{array}{cc} \delta_{c,t}= & \delta_{c,u},\delta_{t,u}=\min_{\varphi_{c,u},\varphi_{t,u}}\frac{1}{2}∥B_{i}-\sum_{u=1}^{U_{c}}h_{c,u}*\varphi_{c,u}-\sum_{u=1}^{U_{t}}h_{t,u}*\varphi_{t,u}{∥}_{2}^{2}\\ \; & \;+\nu_{c}\sum_{u=1}^{U_{c}}\left|\right|\varphi_{c,u}{\left|\right|}_{1}+\nu_{t}\sum_{u=1}^{U_{t}}\left|\right|\varphi_{t,u}{\left|\right|}_{1}, \end{array}$$

where $q_{c}$ = ${\left\{h_{c,u}\right\}}_{u=1}^{U_{c}}$ and $q_{t}$ = ${\left\{h_{t,u}\right\}}_{u=1}^{U_{t}}$ represent the sets of dictionary filters for SR of the cartoon and texture components. $\varphi_{c,u}$ and $\varphi_{t,u}$ are the sparse coefficients that estimate $q_{c}$ and $q_{t}$ when convolved with filters $\left\{h_{c,u}\right\}$ and $\left\{h_{t,u}\right\}$, respectively, and $\nu_{c}$ and $\nu_{t}$ are the positive regularization parameters. The optimization problem is solved iteratively over $\varphi_{c,u}$ and $\varphi_{t,u}$. As, $\varphi_{t,u}$, $h_{t,u}$, and $h_{c,u}$ are fixed, the accompanying issue is settled for the updated $\varphi_{c,u}$ as,

(10) $$\delta_{c,u}=\min_{\varphi_{c,u}}\frac{1}{2}∥A_{i}-\sum_{u=1}^{U_{t}}h_{t,u}*\varphi_{t,u}-\sum_{u=1}^{U_{c}}h_{c,u}*\varphi_{c,u}{∥}_{2}^{2}+\nu_{c}\sum_{u=1}^{U_{c}}\left|\right|\varphi_{c,u}{\left|\right|}_{1}.$$

Similarly, for the updated $\varphi_{t,u}$, keeping $\varphi_{c,u}$ fixed, the accompanying issue is settled as,

(11) $$\delta_{t,u}=\min_{\varphi_{t,u}}\frac{1}{2}∥B_{i}-\sum_{u=1}^{U_{c}}h_{c,u}*\varphi_{c,u}-\sum_{u=1}^{U_{t}}h_{t,u}*\varphi_{t,u}{∥}_{2}^{2}+\nu_{t}\sum_{u=1}^{U_{t}}\left|\right|\varphi_{c,t}{\left|\right|}_{1}.$$

Alternating Direction Method of Multipliers (ADMM)-based CSC [47] is used to address the aforementioned two issues in (10) and (11). This completes the cartoon and texture decomposition phase and allows CSID to proceed to the next phase, which is detailed in the following subsection.

### 3.4. Enhanced CSC-Based Sparse Coding

CSID employs our modified CSC model for cartoon and texture layer decomposition using ${\left\{\varphi_{c,u}\right\}}_{u=1}^{U_{c}}$ and ${\left\{\varphi_{t,u}\right\}}_{u=1}^{U_{t}}$, which represent the sparse coefficient vectors of the cartoon and texture components, respectively. Moreover, the same coefficient vectors are used to evaluate sparse coefficient maps in the next phase of our proposed CSID algorithm, as detailed in the following subsection.

### 3.5. Sparse Coefficient Maps Fusion

CSID applies the l1-norm of the sparse coefficient maps as the activity level measurement of the enhanced images, which remains a common approach adopted in several SR-based image fusion techniques [2,48,49]. Sparse coefficient maps fusion uses an attribute *j* (j∈{c,t}) that refers to the cartoon and the texture components and $\varphi_{j,1:Uj}^{n}\left(p,q\right)$ that uses the $U_{j}$ dimensional vector consisting coefficients of $\varphi_{j,u}^{n}$ at points (p,q). Hence, the initial activity level map $\zeta_{j}^{n}\left(p,q\right)$ map is obtained as,

(12) $$\zeta_{j}^{n}\left(p,q\right)=\left|\right|\varphi_{j,1:U_{j}}^{n}\left(p,q\right){\left|\right|}_{1},j\in \left\{c,t\right\}.$$

A window-based averaging scheme is then applied for noise removal and enhancement of robustness to misregistration. Thus, the activity level map ${\tilde{\zeta }}_{j}^{n}\left(p,q\right)$ is computed as,

(13) $${\tilde{\zeta }}_{j}^{n}\left(p,q\right)=\frac{\sum_{x=-m_{j}}^{m_{n}}\sum_{y=-m_{j}}^{m_{j}}\zeta_{j}^{n}\left(p+x,q+y\right)}{{\left(2m_{j}+1\right)}^{2}},j\in \left\{c,t\right\},$$

where $m_{c}$ and $m_{t}$ refer to the window size for cartoon and texture components, respectively. Finally, CSID employs the “choose-max” rule to obtain the fused coefficient maps ${\left\{\varphi_{j,u}^{f}\right\}}_{u=1}^{U_{j}}$ with j∈{c,t} as,

(14) $$\varphi_{j,1:U_{j}}^{f}\left(p,q\right)=\varphi_{j,1:U_{j}}^{n^{⋄}}\left(p,q\right),n^{⋄}=\arg\max_{n}\left({\tilde{\zeta }}_{j}^{n}\left(p,q\right)\right).$$

This completes the sparse coding phase that leads CSID to the final phase, which is detailed in the following subsection.

### 3.6. Fused Image Reconstruction

This phase is responsible to fuse enhanced CT and MRI images obtained from the aforementioned phases of CSID. The final fused image $A_{f}$ is obtained through the linear combination of ${\left\{\varphi_{c,u}^{f}\right\}}_{u=1}^{U_{c}}$ and ${\left\{\varphi_{t,u}^{f}\right\}}_{u=1}^{U_{t}}$ as,

(15) $$A_{f}=\sum_{u=1}^{U_{c}}h_{c,u}*\varphi_{c,u}^{f}+\sum_{u=1}^{U_{t}}h_{t,u}*\varphi_{t,u}^{f}.$$

This phase completes the multimodal fusion process through our proposed CSID algorithm. The cartoon component includes edges, round and anisotropic structure parts, whereas the texture component contains detailed texture information, periodic behaviors and several levels of noise data. This enables the proposed CSID algorithm to surpass the limitations of the existing fusion techniques (as detailed in Section 2) by allowing the reconstruction of lost information in the CT and MRI images. The reconstruction process results in sharper images with enriched information. The fusion of such enhanced images improves accuracy during clinical diagnosis. The next section discusses the objective evaluation metrics used for the performance evaluation of our proposed CSID algorithm.

## 4. Objective Evaluation Metrics

The objective performance evaluation metrics include mutual information, entropy, feature mutual information, spatial structural similarity, and visual information fidelity, which are used by state-of-the-art works [27,50,51,52,53]. These metrics are defined in the following subsections.

### 4.1. Mutual Information (MI)

MI [50] computes the common information among two discrete variables as follows:

(16) $$MI=\sum_{l=1}^{n}\sum_{m=1}^{n}H_{ij}\left(l,m\right)\log_{2}\frac{H_{ij}\left(l,m\right)}{H_{i}\left(l\right)H_{j}\left(m\right)},$$

where $H_{ij}$ (l,m) denotes the combined probability density distribution of the grayscale image in *i* and *j*. $H_{i}$(*l*) and $H_{j}$(*m*) refer to the probability density distribution of the grayscale image in *i* and *j*, respectively. MI expresses the sum of mutual information between each source image and the fused image. A larger value of MI refers to increased information extracted from the input source images.

### 4.2. Entropy (EN)

EN [27] refers to the measure of information randomness in a sample, which is expressed as,

(17) $$EN\left(x\right)=-\sum_{l=0}^{N-1}H_{i}\left(l\right)\log_{2}H_{i}\left(l\right),$$

where *N* is the number of gray levels, which is taken as 256 in this work, and $H_{i}$(*l*) is the normalized histogram of the fused image *i*.

### 4.3. Feature Mutual Information (FMI)

FMI [51] computes the feature mutual information. A non-reference performance metric for fusion methods is determined as,

(18) $$FMI_{m}^{i,j}=\frac{1}{N}\sum_{l=1}^{N}\frac{I_{l}\left(m,i\right)}{S_{l}\left(m\right)S_{l}\left(i\right)}+\frac{I_{l}\left(m,j\right)}{S_{l}\left(m\right)+S_{l}\left(j\right)},$$

where *N* represents the number of sliding windows, $S_{l}$(*m*) is the entropy of the *n*th window in an image *m*, $I_{l}$(m,i) refers to the regional common information between the *n*th window of images *m* and *i*. Similarly, $I_{l}$(m,j) is the regional MI between the *n*th window of images *m* and *j*. $FMI_{m}^{i,j}$ indicates the amount of edge information transmitted into the fused image from the source images. Here, $FMI_{m}^{i,j}$ remains proportional to the image quality, i.e., a greater value of $FMI_{m}^{i,j}$ yields better quality fused image.

### 4.4. Spatial Structural Similarity (SSS) $Q^{AB/F}$

$Q^{AB/F}$ SSS [52] is an edge-based fusion quality evaluation metric, which determines the quantity of transmitted edge information into the fused image from input images. $Q^{AB/F}$ for a set of source images is computed as,

(19) $$Q^{AB/F}=\frac{\sum_{l=1}^{m}\sum_{j=1}^{n}(Q^{AB}\left(i,j\right)W^{A}\left(i,j\right)+Q^{BF}\left(i,j\right)W^{B}\left(i,j)\right)}{\sum_{l=1}^{m}\sum_{j=1}^{n}(W^{A}\left(i,j\right)+W^{B}\left(i,j)\right)},$$

where $Q^{AB/F}$ (i,j) denotes the information transferred from a source image into the fused image for the pixel location (i,j) and $W^{B}$(i,j) is the weight for a pixel location (i,j). Here, pixels with a higher gradient value influence $Q^{AB/F}$ more in comparison with pixels having a lower gradient value. Thus, $W^{A}$(i,j) = ${\left[Grad\left(x,y\right)\right]}^{T}$: *T* remains constant.

### 4.5. Visual Information Fidelity (VIF)

VIF [53], being a perceptual distortion metric, stands as an important index for image quality assessment. In the context of image fusion, VIF evaluates the performance by calculating common data between a source image and its corresponding fused image. Since VIF provides accurate distortions identification, this work takes average VIF value for the performance evaluation of the given set of algorithms, as shall be discussed in Section 5.

## 5. Performance Evaluation

We evaluate and compare the proposed CSID algorithm to Discrete Wavelet Transform (DWT) [18], Dual Tree Complex Wavelet Transform (DTCWT) [54], Laplacian Pyramid (LP) [21], Guided Filtering based Fusion (GFF) [22], Non-Subsampled Contourlet Transform (NSCT) [19], Non-Subsampled Shearlet Transform domain-Parameter Adaptive Pulse Coupled Neural Network (NSST-PAPCNN) [55], Convolutional Sparse Representation (CSR) [23], Convolutional Sparsity based Morphological Component Analysis (CSMCA) [24], and Convolutional Neural Network (CNN) [25]. The following subsection details the simulation parameters used in this paper.

### 5.1. Simulation Setup

Simulation results are derived using MATLAB R2020b (MathWorks Inc., MA, USA), which is used by state-of-the-art methods for multimodal image fusion due to its extensive built-in libraries support [2,23,24,25]. The hardware platform includes Intel Core i7−9750H 2.59 Giga Hertz processor with 16 GB memory running Microsoft Windows 10 (Microsoft, WA, USA). The multimodal brain image datasets (Data-1 through Data-6) are obtained from [56], which are composed of the CT and MR images. For performance evaluation, selected 500 grayscale images are taken from each of the aforementioned datasets. Input images dimensions are standardized as 256 × 256 pixels. Both qualitative and quantitative analysis are performed for the performance evaluation that are detailed in the following subsections.

### 5.2. Results and Discussion

**Figure 4:**
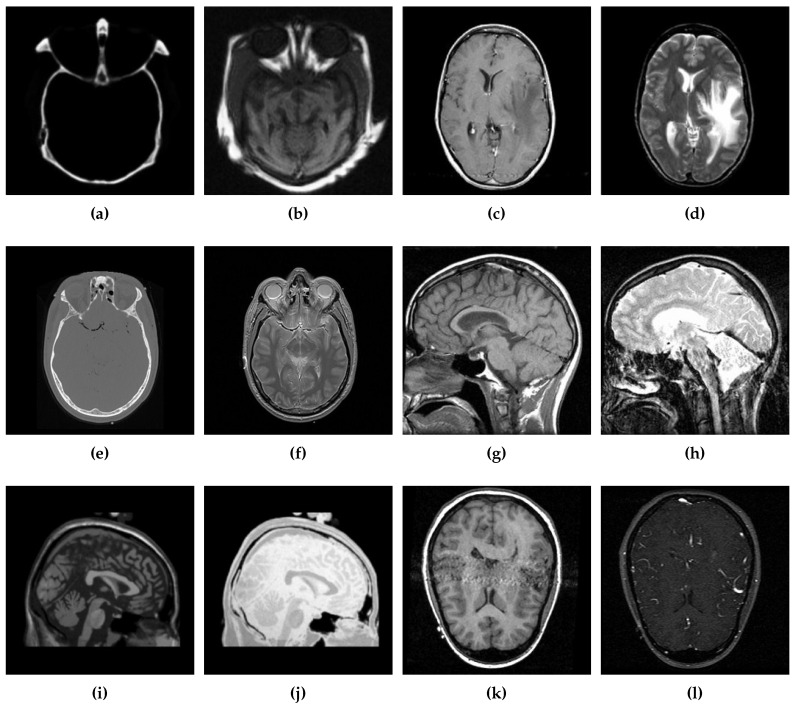
Sample source images from the given datasets (Data-1 through Data-6), where (**a**,**b**) ∈ Data-1, (**c**,**d**) ∈ Data-2, (**e**,**f**) ∈ Data-3, (**g**,**h**) ∈ Data-4, (**i**,**j**) ∈ Data-5, and (**k**,**l**) ∈ Data-6.

**Figure 5:**
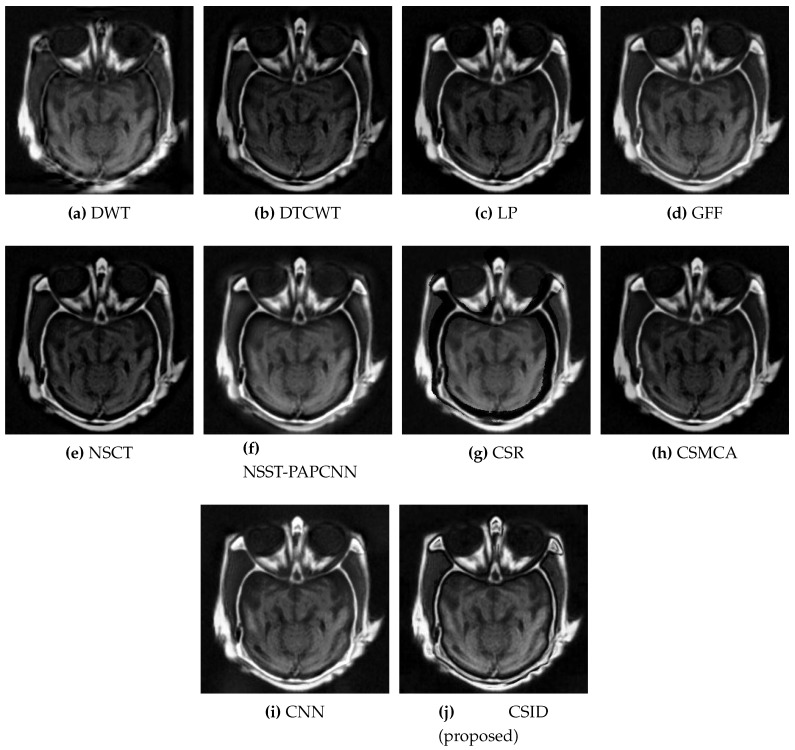
Comparative analysis, based upon visual observation, of the resultant fused images generated by the given set of algorithms using Data-1 dataset.

**Figure 6:**
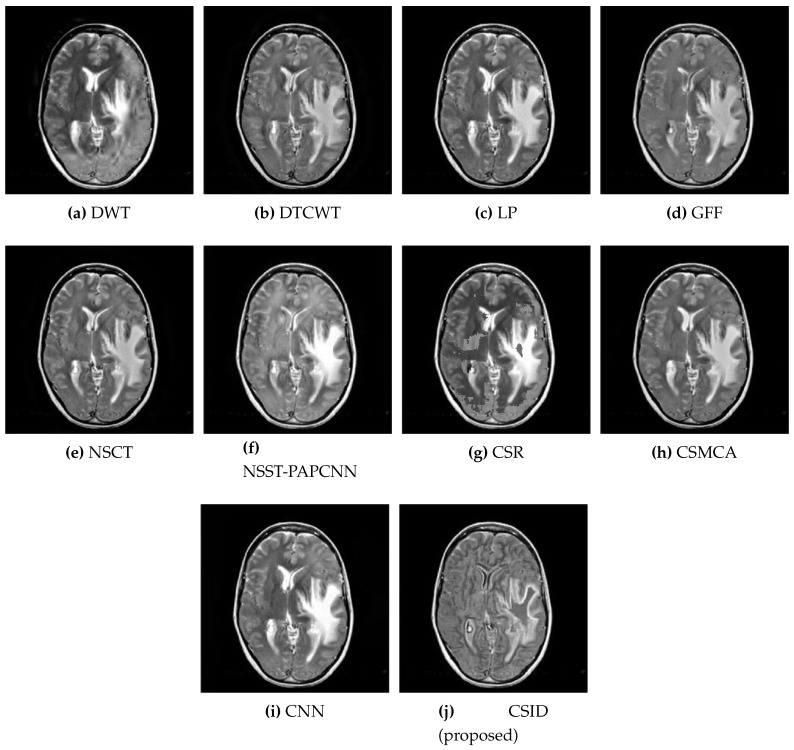
Comparative analysis, based upon visual observation, of the resultant fused images generated by the given set of algorithms using Data-2 dataset.

**Figure 7:**
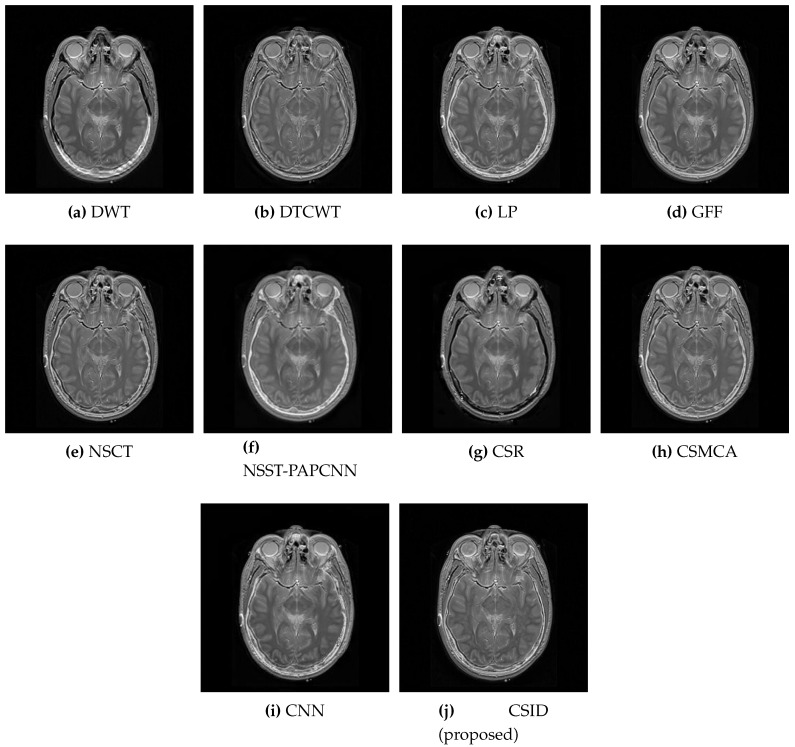
Comparative analysis, based upon visual observation, of the resultant fused images generated by the given set of algorithms using Data-3 dataset.

**Figure 8:**
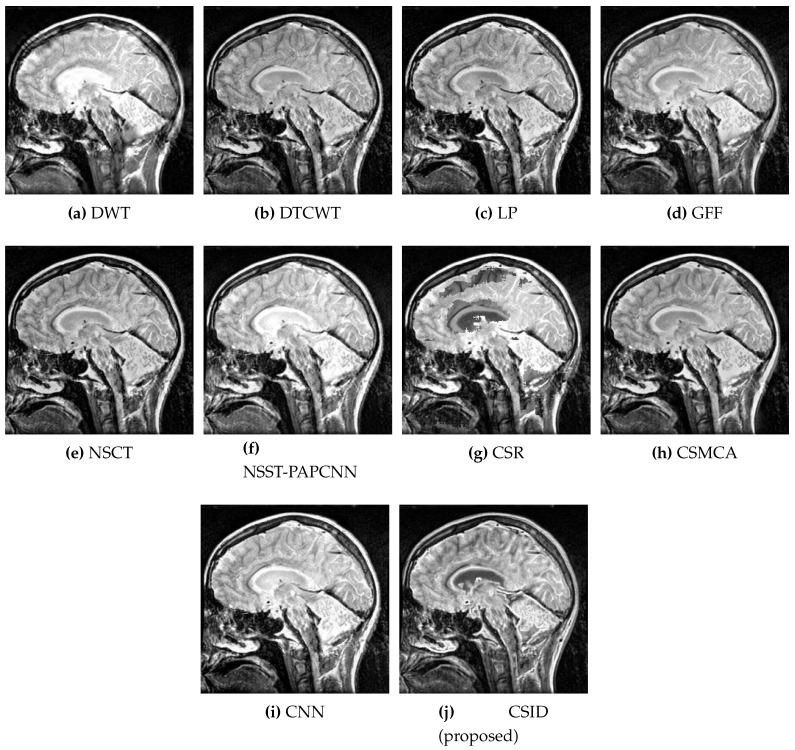
Comparative analysis, based upon visual observation, of the resultant fused images generated by the given set of algorithms using Data-4 dataset.

**Figure 9:**
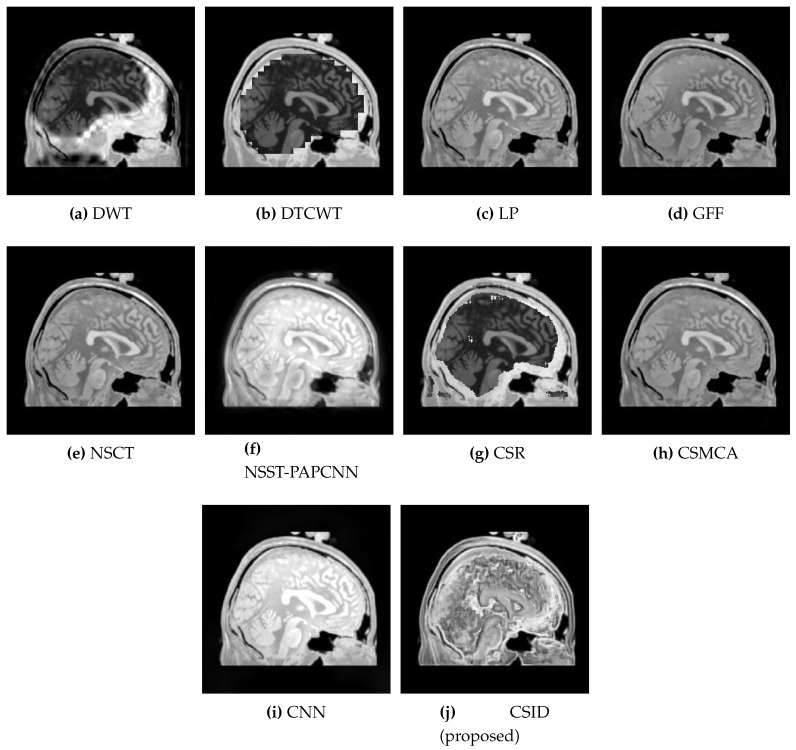
Comparative analysis, based upon visual observation, of the resultant fused images generated by the given set of algorithms using Data-5 dataset.

**Figure 10:**
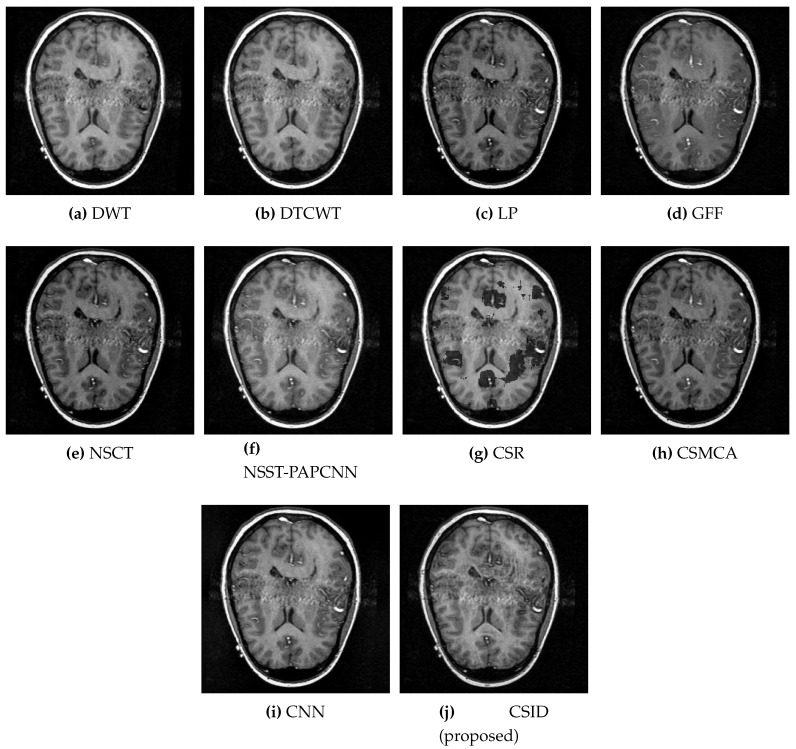
Comparative analysis, based upon visual observation, of the resultant fused images generated by the given set of algorithms using Data-6 dataset.

Six different datasets of multimodal images, referred as Data-1 through Data-6, are used in the simulations. Figure 4 depicts sample images from the aforementioned datasets. The fusion results, generated by our proposed CSID algorithm and the aforementioned eminent fusion algorithms, are shown in Figure 5, Figure 6, Figure 7, Figure 8, Figure 9 and Figure 10. Each result presented is averaged over 20 replicated simulation runs by keeping all the parameters fixed and changing the random seed values. The following subsections demonstrate and discuss the obtained results.

#### 5.2.1. Qualitative Analysis of the Given Set of Algorithms for Multimodal Fusion

This subsection presents the results based on visual observations of the images generated through our proposed CSID algorithm in comparison with the aforementioned algorithms using different datasets, i.e., Data-1 through Data-6. Visual quality comparison of the Data-1 dataset using different fusion methods, i.e., DWT, DTCWT, LP, GFF, NSCT, NSST-PAPCNN, CSR, CSMCA, CNN, and the proposed algorithm are shown in Figure 5a through Figure 5j, respectively. A CT image gives information about hard tissues and their structures, whereas an MRI image indicates information regarding soft tissues. For better diagnosis, it is essential to merge critical information of the aforementioned images into one fused image [12]. In this regard, the aforementioned set of algorithms perform multimodal image fusion. The qualitative results shown in Figure 5 depict inferior performance, in terms of contrast and visual effect, for DWT (Figure 5a), DTCWT (Figure 5b), NSCT (Figure 5e), and CSR (Figure 5g). Note that these algorithms are not capable of preserving information in the fused image, which relates to the objective evaluation metric MI that remains proportional to the level of information extraction. Additionally, Section 5.2.2 further validates this claim through quantitative analysis, where DWT, DTCWT, NSCT, and CSR exhibit lower MI score in comparison with other algorithms. Moreover, GFF (Figure 5d) and NSST-PAPCNN (Figure 5f), yield better results, when compared with DWT, DTCWT, NSCT, and CSR algorithms, by avoiding information loss. However, the lack of noise removal results in over enhancement of the structural features in these algorithms. CSMCA (Figure 5h) and CNN (Figure 5i) further improve the visual quality, where enhanced visualization remains an outcome of lesser information loss. Finally, our proposed CSID algorithm (Figure 5j) yields clear, high contrast and superior visual quality and preserves the salient features, which include considerably enhanced bone structure and soft tissues information in comparison with other given algorithms.

Similarly, Figure 6 shows the qualitative visual analysis for the Data-2 dataset. As a result of the smaller MI score, DWT (Figure 6a), NSCT (Figure 6e), and CSR (Figure 6g) show the highest information loss, among the selected algorithms, for the overlapping areas in MRI and CT images that result in visual deformations of the fused images. Similarly, CNN (Figure 6i) also does not remain effective in transferring information from the source images. The following section (Section 5.2.2) provides quantitative analysis with respect to the given objective evaluation metrics (as discussed in Section 4) that affirms the aforementioned statements. Moreover, in addition to MI, $FMI_{m}^{x,y}$ and $Q^{AB/F}$ evaluation metrics also remain critical that relates to accuracy in the resultant fused images. Although, GFF, NSST-PAPCNN, and CSMCA provide better results in comparison with DWT, NSCT and CSR by conveying complementary information into the fused image, but these algorithms lack accuracy (as shall be discussed in Section 5.2.2 through quantitative analysis). In the end, note that our proposed algorithm (Figure 6j) provides better visual effects in comparison with the other aforementioned algorithms, due to its improved information extraction and edge detection abilities.

Moreover, the qualitative results for the Data-3 dataset are demonstrated in Figure 7. Visual results of DWT (Figure 7a), DTCWT (Figure 7b), LP (Figure 7c), CSR (Figure 7g), and CSMCA (Figure 7h), face issues in edge regions, as these algorithms do not effectively preserve information from the source images. The visual quality of GFF (Figure 7d), NSCT (Figure 7e), NSST-PAPCNN (Figure 7f), and CNN (Figure 7i) remain slightly better, however, these algorithms still remain ineffective to reduce the information loss considerably. Furthermore, the results of our proposed CSID algorithm (Figure 7j) are found the best among all the aforementioned algorithms, as it efficiently preserves edges and texture details.

Image fusion performance is further evaluated using the Data-4 dataset for all the aforementioned algorithms and the corresponding qualitative results are shown in Figure 8. Some image fusion algorithms, such as DWT (Figure 8a), DTCWT (Figure 8b), GFF (Figure 8d), NSST-PAPCNN (Figure 8f), and CSMCA (Figure 8h), show smaller $Q^{AB/F}$ scores that impact sharpness of the resultant fused images. Moreover, these algorithms also experience distorted regions due to lower VIF scores in comparison with other algorithms, as shall be discussed in the quantitative analysis performed in Section 5.2.2. Additionally, LP (Figure 8c) and CSR (Figure 8g) are also found incapable of retaining originality due to increased information loss. NSCT (Figure 8e) and CNN (Figure 8i) provide comparatively improved results, as these algorithms provide effective information integration. Here again, CSID accomplishes the best performance in comparison with the aforementioned algorithms, as it remains capable of transferring more details and provides better contrast.

Furthermore, the aforementioned set of algorithms is evaluated using the Data-5 dataset and the corresponding qualitative results are shown in Figure 9.

Here, DWT (Figure 9a), DTCWT (Figure 9b), NSCT (Figure 9e), NSST-PAPCNN (Figure 9f), and CSR (Figure 9g) provide limited structural information. Moreover, LP (Figure 9c), GFF (Figure 9d), CSMCA (Figure 9h), and CNN (Figure 9i) experience faded edges. However, our proposed CSID algorithm (Figure 9j) shows the superior performance, in comparison with the other aforementioned algorithms, due to its enhanced energy information preservation.

Finally, CSID and the aforementioned set of algorithms are evaluated using the Data-6 dataset, where Figure 10 demonstrates the corresponding qualitative results. All the algorithms, other than CSID, are unable of extracting detailed information that results in blurred fused images. To this end, our proposed CSID algorithm shows improved edge detection and provides enhanced contrast, in comparison with all the aforementioned algorithms that yield better visualization.

#### 5.2.2. Quantitative Analysis of the Given Set of Algorithms for Multimodal Fusion

Table 1 and Table 2 show the results obtained from DWT, DTCWT, LP, GFF, NSCT, NSST-PAPCNN, CSR, CSMCA, CNN, and CSID against the objective metrics, such as MI, EN, FMI, $Q^{AB/F}$, and VIF (as detailed in Section 4). Scores obtained for these metrics remain proportional to the quality of the resultant fused image. Therefore, a smaller score indicates missing information and false structured artifacts during fusion process, whereas a higher score results in enhanced fused images. To this end, all the highest scores obtained are highlighted in bold in Table 1 and Table 2. These results demonstrate that our proposed CSID achieves higher MI, EN, FMI, $Q^{AB/F}$, and VIF scores in comparison with all the other image fusion algorithms using different datasets, i.e., Data-1 through Data-6. This indicates improved performance for CSID, as it remain capable of extracting enriched information from the input source images, thereby, preserving enhanced edges details and yields enhanced visual quality.

In the past few decades, non-invasive applications (like multimodal fusion) have gained tremendous popularity among the healthcare professionals that adds ease and accuracy to the diagnostic process [57,58]. CSID aims to enhance clinical diagnostics by improving the multimodal fusion. We acquired the expert opinion of two healthcare professionals (one radiologist and one physician, whose help we kindly acknowledge) based upon the visual observation of the resultant fused images generated through the given set of algorithms. These experts appreciated the enhanced results generated by CSID in comparison with other state-of-the-art algorithms. Furthermore, it was added by the experts that CSID enables detailed information extraction along with clearer edge detection to yield enhanced fused images, which remain promising for better clinical diagnosis.

#### 5.2.3. Statistical Analysis of the Results

**Figure 11:**
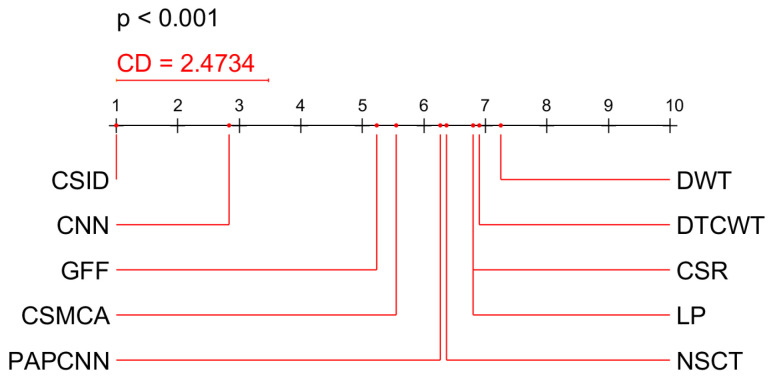
Results of Nemenyi test on different scores evaluating data fusion methods while using six datasets (Data-1 through Data-6). CSID is the proposed method.

We used the non-parametric Friedman’s test and the post-hoc Nemenyi test to analyze how the analyzed methods differ from each other. The Nemenyi test calculates a Critical Difference (CD) using the Tukey’s distribution, and any difference in the ranks between method ranks that is greater than the CD is considered as significantly different [59]. In Figure 11, we used the values from Table 1 and Table 2 to calculate average data fusion method ranks. The results of the Nemenyi test show that the proposed CSID method achieved better values than all other methods when evaluated in terms of MI, EN, $FMI_{m}^{x,y}$, $Q^{AB/F}$, and VIF scores for six datasets (from Data-1 to Data-6), however the advantage over the next best method CNN [25] was not statistically significant (difference between mean ranks <2.4734, Friedman’s p<0.001).

#### 5.2.4. Computational Efficiency

This subsection evaluates the computational efficiency of our proposed CSID algorithm in comparison with DWT, DTCWT, LP, GFF, NSCT, NSST-PAPCNN, CSR, CSMCA, and CNN. Table 1 and Table 2 show the execution time (in seconds) for each of the aforementioned algorithms when applied on the given datasets, i.e., Data-1 through Data-6. The results shown in Table 1 and Table 2 demonstrate that LP exhibits the smallest execution time among the aforementioned algorithms, whereas CSMCA bears the highest execution time. Considering our proposed CSID algorithm, it has smaller execution time in comparison with DTCWT, NSST-PAPCNN, CSR, and CSMCA, and higher execution time than DWT, LP, GFF and NSCT. This is because CSID employs the cartoon-texture component gradient-based feature extraction that enhances image visualization, as shown in Section 5.2.1. Since the main aim of this work is to enhance visualization, a tradeoff in terms of slight increase in execution time remains affordable. Moreover, execution time minimization will be taken as future extension of this work.

## 6. Conclusions

Multimodal medical image fusion has gained a firm stature in modern day healthcare systems. There are several fusion algorithms, which merge multiple input source images to extract detailed information that is exploited to enhance the clinical diagnosis. However, these algorithms have several limitations, such as blurring edges during decomposition, excessive information loss that results in false structured artifacts, and high spatial distortion due to inadequate contrast. This work aims to resolve the aforementioned issues and proposes a novel CSID algorithm that performs contrast stretching and identifies edges by using spatial gradient. CSID proposes the use of cartoon-texture decomposition that creates an overcomplete dictionary. Moreover, this work proposes a modification to the legacy convolutional sparse coding method, and employs enhanced decision maps and fusion rule to obtain the final fused image. Simulation results demonstrate that CSID attains improved performance, in terms of visual quality and enriched information extraction, as compared with other known fusion algorithms. Future work will aim on reducing the execution time of CSID to enable rapid image fusion. Furthermore, the extension of CSID to provide applications, such as visible-infrared image, multi-exposure image, and multi-focus image fusions, can also be taken as a future research direction.

## Figures and Tables

**Table 1:** The quantitative comparison of fusion methods. Best values are shown in bold.

Images	Fusion Methods	MI [50]	EN [27]	${\mathrm{FMI}}_{m}^{x,y}$ [51]	$Q^{\mathrm{AB}/F}$ [52]	VIF [53]	Time (s)
	DWT [18]	2.1141	6.1512	0.7654	0.6656	0.4065	3.644
Data-1	DTCWT [54]	2.1044	6.2074	0.8341	0.6454	0.3976	6.645
	LP [21]	2.5508	6.2724	0.7412	0.6321	0.4141	1.699
	GFF [22]	3.4313	6.7971	0.9032	0.7849	0.4864	3.004
	NSCT [19]	2.2087	6.1488	0.7612	0.6872	0.3864	2.100
	NSST-PAPCNN [55]	2.4665	6.9551	0.4559	0.6968	0.9015	5.083
	CSR [23]	2.087	6.4871	0.3712	0.6327	0.8041	24.037
	CSMCA [24]	2.5863	6.3274	0.4751	0.7373	0.9088	76.700
	CNN [25]	3.5248	6.7541	0.7712	0.7992	0.8991	10.696
	Proposed CSID	**3.9649**	**6.9971**	**0.9781**	**0.8021**	**0.9897**	4.065
	DWT [18]	3.5472	5.5481	0.8493	0.6922	0.5593	3.649
Data-2	DTCWT [54]	3.5201	6.2074	0.8341	0.6756	0.5521	6.544
	LP [21]	3.5908	5.6692	0.8568	0.6571	0.4352	1.783
	GFF [22]	3.8595	5.8459	0.8596	0.5919	0.4295	3.024
	NSCT [19]	3.5110	5.5703	0.8498	0.6837	0.5435	2.112
	NSST-PAPCNN [55]	3.5462	7.7278	0.5597	0.5136	0.8393	5.144
	CSR [23]	3.8744	6.0867	0.5614	0.6667	0.4715	23.441
	CSMCA [24]	3.5008	7.6182	0.5728	0.5772	0.8615	74.994
	CNN [25]	4.2014	7.8421	0.7458	0.6969	0.8015	10.447
	Proposed CSID	**4.8821**	**8.0142**	**0.8850**	**0.7199**	**0.8715**	4.051
	DWT [18]	3.0523	7.1581	0.9438	0.7542	0.5369	3.702
Data-3	DTCWT [54]	3.0871	7.1287	0.9361	0.7414	0.5348	6.414
	LP [21]	3.1847	7.0536	0.8914	0.7499	0.4832	1.955
	GFF [22]	4.0609	5.2463	0.9013	0.6788	0.4486	3.287
	NSCT [19]	3.7394	7.1873	0.9197	0.7101	0.5132	2.089
	NSST-PAPCNN [55]	3.7147	5.3329	0.5536	0.5956	0.8825	5.090
	CSR [23]	3.9478	5.0398	0.8657	0.6342	0.7226	23.339
	CSMCA [24]	3.3098	5.0064	0.4679	0.6397	0.9048	76.018
	CNN [25]	4.0183	6.9420	0.9224	0.7301	0.9755	9.581
	Proposed CSID	**4.4388**	**7.5970**	**0.9744**	**0.7842**	**0.9891**	4.049

**Table 2:** The quantitative comparison of fusion methods. Best values are shown in bold.

Images	Fusion Methods	MI [50]	EN [27]	${\mathrm{FMI}}_{m}^{x,y}$ [51]	$Q^{\mathrm{AB}/F}$ [52]	VIF [53]	Time (s)
	DWT [18]	3.5962	4.7393	0.3823	0.5835	0.9027	3.645
Data-4	DTCWT [54]	3.6632	4.8551	0.8339	0.6921	0.6679	6.643
	LP [21]	3.4733	4.6547	0.7690	0.6391	0.9255	1.774
	GFF [22]	3.4514	4.4081	0.9047	0.6470	0.4961	3.132
	NSCT [19]	3.8544	4.5360	0.8395	0.7093	0.7769	2.143
	NSST-PAPCNN [55]	3.3372	5.0598	0.5401	0.6076	0.8960	5.232
	CSR [23]	3.6584	4.7695	0.8471	0.6655	0.8467	22.998
	CSMCA [24]	3.4007	4.3896	0.4939	0.6601	0.9027	75.802
	CNN [25]	4.2540	5.1748	0.8421	0.7441	0.9408	10.113
	Proposed CSID	**4.6987**	**5.9459**	**0.9814**	**0.8023**	**0.9947**	4.122
	DWT [18]	4.0214	4.6386	0.4777	0.5782	0.7592	3.650
Data-5	DTCWT [54]	4.2985	4.7687	0.4885	0.6257	0.5573	6.625
	LP [21]	4.4128	4.8825	0.5241	0.6825	0.5826	1.874
	GFF [22]	4.7093	5.2982	0.7849	0.7259	0.7928	3.332
	NSCT [19]	3.9309	4.9304	0.6908	0.6827	0.7469	2.139
	NSST-PAPCNN [55]	4.1937	4.9809	0.7360	0.6887	0.6993	5.403
	CSR [23]	4.5094	5.0297	0.6997	0.6259	0.5067	23.422
	CSMCA [24]	5.0924	5.9330	0.7485	0.7759	0.8257	76.112
	CNN [25]	5.1118	5.9989	0.8697	0.8267	0.8881	10.691
	Proposed CSID	**5.2471**	**6.2874**	**0.8847**	**0.8728**	**0.8971**	4.041
	DWT [18]	3.6877	4.8474	0.5570	0.4938	0.5551	3.647
Data-6	DTCWT [54]	3.6439	4.8839	0.5683	0.5097	0.6086	6.245
	LP [21]	3.9482	4.9029	0.6019	0.6287	0.6239	1.963
	GFF [22]	4.1675	5.0098	0.7829	0.6876	0.7452	3.504
	NSCT [19]	3.8888	4.8729	0.7067	0.6431	0.7884	2.146
	NSST-PAPCNN [55]	4.0671	4.9038	0.7149	0.6835	0.7763	5.113
	CSR [23]	3.7432	4.4597	0.6839	0.5334	0.6720	23.483
	CSMCA [24]	4.5810	4.9997	0.8097	0.7482	0.8027	76.772
	CNN [25]	4.6744	5.2779	0.8527	0.7983	0.8341	10.834
	Proposed CSID	**4.8887**	**5.8209**	**0.8817**	**0.8497**	**0.8748**	4.047

## References

[1] Liu X., Liu Q., Wang Y. (2020). Remote sensing image fusion based on two-stream fusion network. Inf. Fusion.

[2] Maqsood S., Javed U. (2020). Biomedical Signal Processing and Control Multi-modal Medical Image Fusion based on Two-scale Image Decomposition and Sparse Representation. Biomed. Signal Process. Control.

[3] Chen G., Li C., Wei W., Jing W., Woźniak M., Blažauskas T., Damaševičius R. (2019). Fully convolutional neural network with augmented atrous spatial pyramid pool and fully connected fusion path for high resolution remote sensing image segmentation. Appl. Sci..

[4] Nisa M., Shah J.H., Kanwal S., Raza M., Khan M.A., Damaševičius R., Blažauskas T. (2020). Hybrid malware classification method using segmentation-based fractal texture analysis and deep convolution neural network features. Appl. Sci..

[5] Bernardo L.S., Quezada A., Muñoz R., Maia F.M., Pereira C.R., Wu W., de Albuquerque V.H.C. (2019). Handwritten pattern recognition for early Parkinson’s disease diagnosis. Pattern Recognit. Lett..

[6] Gambhir D., Manchanda M. (2019). Waveatom transform-based multimodal medical image fusion. Signal Image Video Process..

[7] Manchanda M., Sharma R. (2018). An improved multimodal medical image fusion algorithm based on fuzzy transform. J. Vis. Commun. Image Represent..

[8] Ke Q., Zhang J., Wei W., Damaševičius R., Woźniak M. (2019). Adaptive independent subspace analysis of brain magnetic resonance imaging data. IEEE Access.

[9] Wei W., Zhou B., Polap D., Wozniak M. (2019). A regional adaptive variational PDE model for computed tomography image reconstruction. Pattern Recognit..

[10] Guo Z., Li X., Huang H., Guo N., Li Q. (2019). Deep Learning-Based Image Segmentation on Multimodal Medical Imaging. IEEE Trans. Radiat. Plasma Med. Sci..

[11] Khan M.A., Ashraf I., Alhaisoni M., Damaševičius R., Scherer R., Rehman A., Bukhari S.A.C. (2020). Multimodal Brain Tumor Classification Using Deep Learning and Robust Feature Selection: A Machine Learning Application for Radiologists. Diagnostics.

[12] Maqsood S., Javed U., Riaz M.M., Muzammil M., Muhammad F., Kim S. (2020). Multiscale Image Matting Based Multi-Focus Image Fusion Technique. Electronics.

[13] James A.P., Dasarathy B.V. (2014). Medical image fusion: A survey of the state of the art. Inf. Fusion.

[14] Hermessi H., Mourali O., Zagrouba E. (2018). Convolutional neural network-based multimodal image fusion via similarity learning in the shearlet domain. Neural Comput. Appl..

[15] Wang L., Li B., Tian L.F. (2014). Multi-modal medical image fusion using the inter-scale and intra-scale dependencies between image shift-invariant shearlet coefficients. Inf. Fusion.

[16] Li H., Qiu H., Yu Z., Li B. (2017). Multifocus image fusion via fixed window technique of multiscale images and non-local means filtering. Signal Process..

[17] Yang S., Wang M., Jiao L., Wu R., Wang Z. (2010). Image fusion based on a new contourlet packet. Inf. Fusion.

[18] Yang Y. (2011). A novel DWT based multi-focus image fusion method. Procedia Eng..

[19] Li H., Qiu H., Yu Z., Zhang Y. (2016). Infrared and visible image fusion scheme based on NSCT and low-level visual features. Infrared Phys. Technol..

[20] Nencini F., Garzelli A., Baronti S., Alparone L. (2007). Remote sensing image fusion using the curvelet transform. Inf. Fusion.

[21] Du J., Li W., Xiao B. (2016). Union laplacian pyramid with multiple features for medical image fusion. Neurocomputing.

[22] Li S., Kang X., Hu J. (2013). Image fusion with guided filtering. IEEE Trans. Image Process..

[23] Liu Y., Chen X., Ward R.K., Wang Z.J. (2016). Image Fusion With Convolutional Sparse Representation. IEEE Signal Process. Lett..

[24] Liu Y., Chen X., Ward R.K., Wang Z.J. (2019). Medical Image Fusion via Convolutional Sparsity Based Morphological Component Analysis. IEEE Signal Process. Lett..

[25] Liu Y., Chen X., Cheng J., Peng H. A medical image fusion method based on convolutional neural networks. Proceedings of the 2017 20th International Conference on Information Fusion (Fusion).

[26] Yang B., Li S. (2014). Visual attention guided image fusion with sparse representation. Optik (Stuttg)..

[27] Liu Y., Liu S., Wang Z. (2015). A general framework for image fusion based on multi-scale transform and sparse representation. Inf. Fusion.

[28] Li S., Yin H., Fang L. (2012). Group-sparse representation with dictionary learning for medical image denoising and fusion. IEEE Trans. Biomed. Eng..

[29] Kim M., Han D.K., Ko H. (2016). Joint patch clustering-based dictionary learning for multimodal image fusion. Inf. Fusion.

[30] Basar S., Adnan A., Khan N.H., Haider S. Color image segmentation using K-mean classification on RGB histrogram. Proceedings of the Recent Advances In Telecommunications, Informatics And Educational Technologies.

[31] Litjens G., Kooi T., Bejnordi B.E., Setio A., Ciompi F., Ghafoorian M. (2017). A survey on deep learning in medical image analysis. Med. Image Anal..

[32] Wang K., Zheng M., Wei H., Qi G., Li Y. (2020). Multi-modality medical image fusion using convolutional neural network and contrast pyramid. Sensors.

[33] Zhang Q., Levine M.D. (2016). Robust multi-focus image fusion using multi-task sparse representation and spatial context. IEEE Trans. Image Process..

[34] Xing C., Wang M., Dong C., Duan C., Wang Z. (2020). Using Taylor Expansion and Convolutional Sparse Representation for Image Fusion. Neurocomputing.

[35] Li Y., Sun Y., Huang X., Qi G., Zheng M., Zhu Z. (2018). An image fusion method based on sparse representation and Sum Modified-Laplacian in NSCT Domain. Entropy.

[36] Li W., Jia L., Du J. (2019). Multi-Modal Sensor Medical Image Fusion Based on Multiple Salient Features with Guided Image Filter. IEEE Access.

[37] Arif M., Wang G. (2020). Fast curvelet transform through genetic algorithm for multimodal medical image fusion. Soft Comput..

[38] Kaur M., Singh D. (2020). Fusion of medical images using deep belief network. Cluster Comput..

[39] Shahdoosti H.R., Mehrabi A. (2018). Multimodal image fusion using sparse representation classification in tetrolet domain. Digital Signal Process..

[40] Ying Z., Li G., Gao W. (2017). A Bio-Inspired Multi-Exposure Fusion Framework for Low-light Image Enhancement. arXiv.

[41] Yan J., Li J., Fu X. (2019). No-Reference Quality Assessment of Contrast-Distorted Images using Contrast Enhancement. arXiv.

[42] Poddar S., Tewary S., Sharma D., Karar V., Ghosh A., Pal S.K. (2013). Non-parametric modified histogram equalisation for contrast enhancement. IET Image Process..

[43] Schindelin J., Rueden C.T., Hiner M.C., Eliceiri K.W. (2015). The ImageJ ecosystem: An open platform for biomedical image analysis. Mol. Reprod. Dev..

[44] Pinheiro P.O., Collobert R. From image-level to pixel-level labeling with convolutional networks. Proceedings of the 28th IEEE conference on computer vision and pattern recognition, CVPR 2015.

[45] Gao W., Zhang X., Yang L., Liu H. An improved Sobel edge detection. Proceedings of the 3rd International Conference on Computer Science and Information Technology.

[46] Zhang H., Patel V.M. (2017). Convolutional sparse and low-rank coding-based image decomposition. IEEE Trans. Image Process..

[47] Wohlberg B. (2016). Efficient algorithms for convolutional sparse representation. IEEE Trans. Image Process..

[48] Yang B., Li S. (2012). Pixel-level image fusion with simultaneous orthogonal matching pursuit. Inf. Fusion.

[49] Jiang Y., Wang M. (2014). Image fusion with morphological component analysis. Inf. Fusion.

[50] Hossny M., Nahavandi S., Vreighton D. (2008). Comments on information measure for performance of image fusion. Electron. Lett..

[51] Haghighat M.B.A., Aghagolzadeh A., Seyedarabi H. (2011). A non-reference image fusion metric based on mutual information of image features. Comput. Electr. Eng..

[52] Petrovi V.S., Xydeas C.S. (2003). Sensor noise effects on signal-level image fusion performance. Inf. Fusion.

[53] Han Y., Cai Y., Cao Y., Xu X. (2013). A new image fusion performance metric based on visual information fidelity. Inf. Fusion.

[54] Yu B., Jia B., Ding L., Cai Z., Wu Q. (2016). Hybrid dual-tree complex wavelet transform and support vector machine for digital multi-focus image fusion. Neurocomputing.

[55] Yin M., Liu X., Liu Y., Chen X. (2019). Medical Image Fusion With Parameter-Adaptive Pulse Coupled-Neural Network in Nonsubsampled Shearlet Transform Domain. IEEE Trans. Instrum. Measur..

[56] Zhu Z., Chai Y., Yin H., Li Y., Liu Z. (2016). A novel dictionary learning approach for multi-modality medical image fusion. Neurocomputing.

[57] Madanagopal R. (2014). Medical fusion imaging: Paving the way for better diagnosis of tumours. Health Manag..

[58] Amini N., Fatemizadeh E., Behnam H. (2014). MRI-PET image fusion based on NSCT transform using local energy and local variance fusion rules. J. Med. Eng. Technol..

[59] Demsar J. (2006). Statistical comparisons of classifiers over multiple data sets. J. Mach. Learn. Res..

